# Changes in Stem Cell Regulation and Epithelial Organisation during Carcinogenesis and Disease Progression in Gynaecological Malignancies

**DOI:** 10.3390/cancers13133349

**Published:** 2021-07-03

**Authors:** Paula Cunnea, Christina Fotopoulou, Jennifer Ploski, Fabian Trillsch, Sven Mahner, Mirjana Kessler

**Affiliations:** 1 Division of Cancer, Department of Surgery and Cancer, Faculty of Medicine, Imperial College London, London W12 0NN, UK; p.cunnea@imperial.ac.uk (P.C.); c.fotopoulou@imperial.ac.uk (C.F.); j.ploski@imperial.ac.uk (J.P.); 2 Department of Obstetrics and Gynaecology, University Hospital, LMU Munich, Marchioninistr 15, 81377 Munich, Germany; Fabian.trillsch@med.uni-muenchen.de (F.T.); Sven.Mahner@med.uni-muenchen.de (S.M.)

**Keywords:** ovarian cancer, endometrial cancer, cervical cancer, patient-derived organoids, cancer stem cells, Wnt signalling, epithelial differentiation, TP53, HDR mechanisms, BRCA1/2

## Abstract

**Simple Summary:**

Recent advances in our understanding of the stem cell potential in adult tissues have far-reaching implications for cancer research, and this creates new opportunities for the development of new therapeutic strategies. Here we outline changes in stem cell biology that characterize main gynaecological malignancies, ovarian, endometrial, and cervical cancer, and focus on specific differences between them. We highlight the importance of the local niche environment as a driver of malignant transformation in addition to mutations in key cancer-driving genes. Patient-derived organoids capture in vitro main aspects of cancer tissue architecture and stemness regulatory mechanisms, thus providing a valuable new platform for a personalized approach in the treatment of gynecological malignancies. This review summarizes the main achievement and formulates remaining open questions in this fast-evolving research field.

**Abstract:**

Gynaecological malignancies represent a heterogeneous group of neoplasms with vastly different aetiology, risk factors, molecular drivers, and disease outcomes. From HPV-driven cervical cancer where early screening and molecular diagnostics efficiently reduced the number of advanced-stage diagnosis, prevalent and relatively well-treated endometrial cancers, to highly aggressive and mostly lethal high-grade serous ovarian cancer, malignancies of the female genital tract have unique presentations and distinct cell biology features. Recent discoveries of stem cell regulatory mechanisms, development of organoid cultures, and NGS analysis have provided valuable insights into the basic biology of these cancers that could help advance new-targeted therapeutic approaches. This review revisits new findings on stemness and differentiation, considering main challenges and open questions. We focus on the role of stem cell niche and tumour microenvironment in early and metastatic stages of the disease progression and highlight the potential of patient-derived organoid models to study key events in tumour evolution, the appearance of resistance mechanisms, and as screening tools to enable personalisation of drug treatments.

## 1. Introduction

Gynaecological malignancies represent a great medical burden for women’s health in general. Together, uterine, cervical, and ovarian cancer are responsible for approximately 30,000 deaths every year in the USA (CDC data) [1], with an average of 10,000 deaths in Germany and similar prevalence also observed in other countries with well-organized health care systems. The incidence of cervical cancer continues to be disproportionally high in developing countries where early screening (Pap test), as well as HPV diagnostics and access to the vaccine, are not readily available. Endometrial cancer occurs more frequently than ovarian cancer, but a significantly shorter long-term survival in the latter case leads to disproportional high mortality, and ovarian cancer accounts for more than 50% of gynaecological cancer deaths [2]. While the overall difference in long-term prognosis is likely the consequence of earlier detection of endometrial cancer due to the early onset of more specific symptoms, substantial differences in the biology of these cancers and their responses to therapy are major factors that determine the course of the disease. The great diversity in molecular, histological, and genetic characteristics between ovarian, uterine, and cervical cancers is particularly intriguing due to all having the same developmental origins as all structures starting with the upper vagina, uterus and fallopian tubes develop from Mullerian ducts, as PAX8+ positive mucosal surfaces of columnar epithelium.

Although the whole genital system functions within the hormonal milieu during reproductive years, in some cases, the same physiological stimuli have opposing effects on cancer risk depending on the cancer type. For example, while oral contraceptives significantly reduce the risk of ovarian and endometrial cancers, they increase the risk for cervical cancer [3,4]. Moreover, obesity and diet are strongly associated with endometrial cancer risk [5] but appear not to play a major role in the development of high-grade serous ovarian cancer (HGSOC) [6]. These facts suggest that there are substantial differences in local homeostatic environments in areas of the genital tract and cancer type-specific combination of autonomous and exogenous factors are required to cause transformation.

## 2. Organisation of the Stemness Compartments in the Genital Tract

### 2.1. Control of Regeneration in Healthy Mucosa

**Figure 1:**
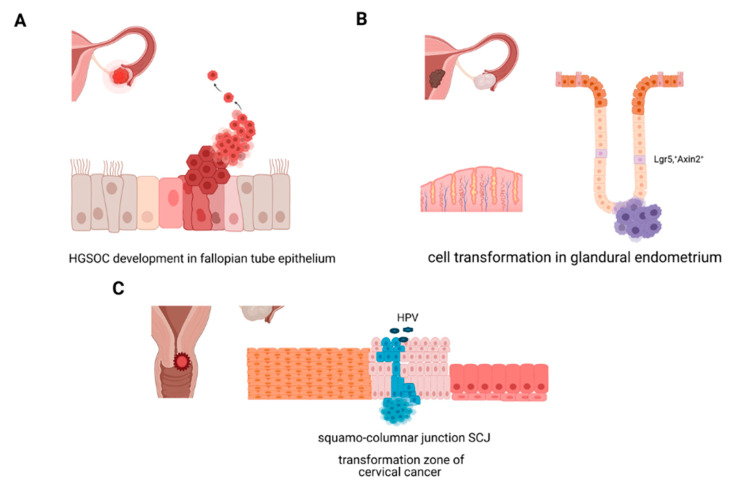
Overview of cell transformation mechanism in ovarian, uterine and cervical cancers. (**A**) HGSOC develops from secretory cells of fallopian tube epithelium. (**B**) Model of endometrial cancer development postulates central role of the stem cells in the uterine glands during the transformation. (**C**) Cervical cancer arises in the transformation zone/squamo-columnar junction of the cervix and is driven by HPV viral infection.

Epithelial homeostasis in healthy tissue is maintained by the presence of long-lived progenitor cells that continuously give rise to differentiated progeny. Wnt pathway signalling gradients play a central role in the maintenance of homeostasis in all structures of Mullerian duct origin: fallopian tubes, uterus, and cervix. Active Wnt signalling is essential for the development of the genital tract, which is apparent in strong defects in female sex determination during embryogenesis caused by deleterious mutations of Wnt pathway components [7]. While core mechanisms of epithelial development are preserved in the adult tissue to ensure continuous cellular turnover within the mucosa, changes in regulatory mechanisms have been identified at different stages of organ maturation. Cyclic exposure to hormonal stimulation as a major driver of the differentiation and functional determinant provides an additional layer of complexity that needs to be understood to identify critical steps of disease development. Wnt signalling receptor Lgr5, a determinant of stemness in many adult tissues, is broadly expressed during embryonic development in all regions of the Mullerian tract. However neonatal mucosa contains only rare Lgr5+ cells at the tip of developing uterine glands of the endometrium while Lgr5+ expression in the adult fallopian tube, and the vaginal epithelium is very weak [8,9]. Nevertheless, it has been clearly shown that active Wnt signalling controls regeneration in all segments of the genital tract starting with the endocervix towards the ovary as fallopian tube, endometrial, and endocervical organoid models all show a degree of dependency on exogenous supplementation of Wnt agonists in the culture media. A lineage tracing study in mouse models identified the hilum, connecting ovary and the fallopian tube as the region of interest where Lgr5+ cells capable of regeneration reside on the surface of the ovary [10]. The mechanisms that control regeneration of the adult fallopian tube epithelium, localization of the adult stem cells and their exact molecular profile have not yet been determined. However, there is a broad consensus that HGSOC develops in the distal fallopian tube epithelium from secretory cells with mutated TP53 signatures, but the molecular mechanism of the transformation process and potential involvement of tissue resident progenitors remains unknown (Figure 1A). The uterus epithelium has extraordinary regeneration capacity, as complete shedding of the endometrial lining occurs at the end of each menstrual cycle. Comprehensive lineage tracing study in a mouse model suggested the existence of two separate stem cell compartments. One compartment consisting of a more active population that guides turnover of the luminal epithelium, and the second comprised of slow cycling Axin2+ stem cells at the bottom of the glands that are functional also in absence of hormonal stimulation and are putative cells of origin of endometrial cancer (Figure 1B) [11].

A recent study in mice demonstrated that the Wnt gradient defines the border between endocervical columnar epithelium and squamous epithelium of the ectocervix, where likely two different populations of stem cells are regenerating epithelial compartments depending on the signalling received from the stroma [12]. While expression of Krt5/Krt14 markers characterize squamous epithelium, columnar lineage exhibits hallmark expression of Krt8/Krt19. These characteristics are also found in two main cervical cancer types squamous cell carcinoma (SCC) and adenocarcinoma (ADC) indicating potential lineage continuity between healthy stem cells and cervical cancer tissue (Figure 1C).

Still, more comprehensive molecular studies are necessary to understand how regeneration processes are regulated on the cellular level in response to different physiological environments in all regions of the genital tract.

### 2.2. Changes in Stemness during Transformation

The intersection between intrinsic and extrinsic factors in tumour development occurs at the level of the stem-cell compartment, which is regulated by paracrine signalling. Epithelium regeneration has an intrinsic lineage hierarchy, but the stromal compartment can produce signals that directly influence stem cell differentiation [13]. Although many cancers are driven by mutational events in key cancer-driving genes, it is becoming increasingly clear that healthy tissue has several protective mechanisms to prevent the outright proliferation of mutated clones and maintain homeostasis. As shown convincingly in the genetic model of mouse skin, activation of β-catenin causes only temporary expansion of mutant clones, which regress within four weeks [14]. In line with this, it is perhaps not surprising that large NGS sequencing studies of colonic crypts revealed regular occurrence of tumour driving mutations in healthy individuals [15], and similar results were obtained following analysis of uterine lavages [16]. Moreover, there is strong evidence from malignancies of the intestinal tract that the local inflammatory microenvironment directly influences changes in the homeostasis of the stem cell compartment and favours the selection of mutated clones [17]. Because TP53 driver mutations are present in nearly all HGSOC patients—a malignancy where development is likely driven by the pro-inflammatory microenvironment associated with the process of ovulation—further investigation of the clonal competition in the distal fallopian tube is of pivotal importance to understand the mechanism of carcinogenesis. In this context it would be also interesting to further explore the role of the pathogens in long term changes of homeostatic conditions in the fallopian tube epithelium [18].

#### 2.2.1. Molecular Origins of Ovarian Cancer

Epithelial ovarian cancer is a heterogeneous disease, classified based on the histologic types on high-grade serous, mucinous, endometrioid, clear cell cancer, and transitional cell carcinoma. While HGSOC likely originates from the epithelium of the distal fallopian tube, the phenotypic characteristics and molecular profile of mucinous tumours indicate that they could be metastasis from the intestinal tract, and endometrioid and clear cell are the rare malignant transformation of endometriotic tissue from the uterus [19].

Secretory cell outgrowth is presumed to be an early step in HGSOC aetiology [20]. Serous tubal intraepithelial carcinoma (STIC) has been identified as early premalignant lesions in the distal fallopian tube that gives rise to HGSOC [21]. Mutational profiling by whole exome sequencing (WES) confirmed continuity in mutational profiles between STICs and cancer tissue [22], but it is yet to be determined if STIC formation is a necessary intermediate step in cancer development [23], and the clinical relevance of isolated STIC detection remains unclear [24].

Lineage tracing studies in mice [25] and temporal analysis of in vitro differentiation of the bipotent epithelial progenitors isolated from the human fallopian tube [26] confirmed that secretory cells are precursors of ciliated cells. Accordingly, the question arises if HGSOC carcinogenesis is the result of the escape from the homeostatic control mechanism that regulates differentiation.

#### 2.2.2. Molecular Origins of Endometrial Cancer

Endometrial cancer is a malignancy of the inner uterine lining and the most frequent gynaecological malignancy in industrialised countries. Endometrial cancer is broadly classified into two types: endometrioid cancer (75% of cases) and serous cancer (25%). Serous endometrial cancer shares some important genetic hallmarks with HGSOC, >90% prevalence of TP53 mutation and high copy number variations (CNV), while the more common endometrioid type has recurring PTEN, PIK3CA, and ARID1A mutations [27]. Conditional expression of oncogenic PIK3CA together with β-catenin activation in genetic mouse models showed that the tumour which develops arises from Axin2+ positive stem cells [11]. While estradiol is the main driver of endometrial proliferation, its involvement in the development of endometrial cancer is not completely understood. This is especially intriguing as estradiol is a major component of the pathophysiology of endometriosis, another major morbidity of the uterine lining.

Endometriosis is a prevalent uterine disease that affects up to 10% of women of active reproductive age and has a debilitating effect on the fertility and quality of life of the patients but is a generally benign condition. Its main manifestation is uncontrolled expansion of endometrial epithelium which can extend to extrauterine sites such as ovary or peritoneum. On the molecular level, it represents a very interesting example of extensive change in phenotype and great proliferative potential of the epithelium but without the invasive biology characteristic of malignancies. Subtle changes in growth requirements of endometrial organoids are reported [28,29], which suggest differences in the involvement of BMP signalling in endometriosis tissue compared to the healthy endometrium. Comparison of transcriptional profiles revealed significant changes in the expression of genes that regulate interactions with the extracellular matrix, differences in activation levels of PI3K and Wnt signalling, and denominators of hormone response. Interestingly, analysis of exome sequencing data from deep infiltrating endometrioid lesions of advanced endometriotic lesions (stage IV) revealed the presence of somatic mutations in tumour driving genes (CRCF, EP300, KRAS, etc.) [30]. These findings further reiterate the importance of broader tissue context and changes in the microenvironment for the understanding of endometrial carcinogenesis.

#### 2.2.3. Molecular Origins of Cervical Cancer: Cellular Transformation in the Cervix, HPV, and Stemness

The cervix connects the uterine cavity with the vagina and consists of the endocervix, composed of a single layer of mucin-secreting columnar cells, and ectocervix, composed of stratified squamous epithelium projecting into the vagina. The squamocolumnar junction (SCJ) is formed from the transition of the columnar epithelium to the squamous epithelium also frequently termed the transformation zone [31]. Cervical cancer is almost exclusively driven by latent chronic HPV infections of the SCJ epithelium. A large TCGA study of 228 cancers confirmed integration of the viral genome in all HPV18 related cases and 76% of HPV16 related cases. Interestingly, HPV-driven cancers had a distinct mutational profile with prominent APOBEC signatures, while HPV negative cancers were predominantly endometrial-like cervical cancers with a mutational profile resembling endometrial cancer (ARID1A, PTEN, and KRAS mutations). Though the central role of E6/E7 oncogenic viral proteins in cellular transformation has been extensively studied, mechanistic connection between carcinogenesis and stem cell mechanism at the transition zone is not completely understood. Analysis of primary isolates enriched with stem cells from cervical cancer identified a positive regulatory mechanism between overexpression of E6 and induction of HES1, one of the main transcription factors of the NOTCH signalling pathway [32]. A recent study successfully established organoid lines from positive Pap test samples from ectocervix and endocervix and demonstrated maintenance of HPV integration in the genome [33]. Accordingly, these organoids displayed in vitro and in vivo phenotypic characteristics of cervical cancer and are useful models for molecular studies of cellular transformation as well as patient-specific drug responses.

### 2.3. Stemness Markers Correlate with Therapy Response

Cancer stem cells (CSCs) have been widely investigated in different malignancies to better understand disease progression and recurrence. Although each cancer type has a unique group of stem cell markers, they share expression of stemness-related genes, notably SOX2, OCT4, and NANOG. These genes are responsible for maintaining the stem cell-like phenotype, including conserving self-renewal capacity and pluripotency.

In endometrial cancer, cancer stem cell populations are characterised by cells expressing CD117 and CD133, both demonstrating increased tumourigenicity and reduced platinum sensitivity [34,35,36]. High expression of these markers is associated with poor overall survival (OS) [34,35]. Furthermore, these markers also represent potential therapeutic targets. Imatinib, a tyrosine kinase inhibitor that targets CD117 (also known as c-kit), has been shown to inhibit cell growth in vitro, as well as working synergistically with cisplatin to inhibit tumour growth in mouse xenografts [34]. CD44 expression was observed in the infiltrative-type myometrial invasion, and also associated with reduced E-cadherin expression, suggesting that the process of epithelial-mesenchymal transition (EMT) is active in this cell population [37]. Identifying these CSC markers associated with aggressive phenotypes would be useful for stratifying patients who may require more robust treatment strategies. CD55 was identified as necessary and sufficient for the maintenance of the CSC population in endometrioid ovarian and endometrial cell lines, where inhibiting CD55 resulted in reduced expression of OCT4, SOX2, and NANOG [38]. CD55 was also found to regulate sensitivity to platinum in cell culture models, as inhibition of CD55 increased response to platinum even in the cisplatin-resistant endometrioid ovarian cell line A2780-CP70 and endometrial cisplatin-resistant HEC1a primary cell line [38].

Analysis of cellular interactions of HPV oncoproteins E6 and E7 with host signalling networks is key for understanding of the progression from high risk HPV infection to cervical carcinoma. It was shown that they cause the degradation of tumour suppressors p53 and retinoblastoma-associated protein, which would normally repress stemness genes OCT4, SOX2, NANOG, and fibroblast growth factor 4 [39,40]. OCT4 and SOX2 expression have been shown to be independent prognostic factors in cervical cancer, with OCT4 associated with poorly differentiated tumours and advanced stage disease [41], and SOX2 associated with poor overall survival and recurrence [42]. ALDH1, a well characterised CSC marker, correlated with poor disease-free survival and overall survival in cervical cancer patients, as well as being an independent predictor of response to chemotherapy [43]. Myeloid-derived suppressor cells in the tumour microenvironment were found to enhance stemness by increasing the ALDH1+ cell population in cervical cancer cell lines [44]. Stem-like cells isolated from primary cervical tumours, capable of self-renewal, spheroid formation, and tumour initiation, were found to have high expression of CD44 and CK17 markers, ABCG2 drug transporter, and stemness related genes OCT4 and SOX2 [45]. Analysis of the TCGA cohort of cervical squamous cell carcinoma and endocervical adenocarcinoma identified over-expression of a subset of stemness-related genes associated with poor prognosis, mostly belonging to the Focal Adhesion pathway [46].

As with other gynaecological cancers, higher expression of CSC markers in ovarian cancer is associated with chemotherapy resistance and poor patient outcomes. A side population of cells expressing NANOG, OCT4 and ABCG2/BCRP1 was isolated from ovarian cancer cell lines and ascites of ovarian cancer patients, and was found to have increased tumourgenicity and chemoresistance [47]. Similar findings of increased tumourigenicity were associated with CD133, ALDH1, EpCAM, and CK7 expression in primary ovarian tumour and ascites samples [48,49] and ovarian cancer cell lines [50]. Knockdown of LGR6 in ovarian cancer cell lines showed improved sensitivity to cisplatin or paclitaxel, reduced spheroid formation, and reduced expression of CSC markers (CD133, SOX2, ALDH1, OCT4, NANOG) [51], thus suggesting a potential target for treatment, as loss of LGR6 led to reduced stemness and reversal of chemoresistance.

Several studies have also demonstrated mesenchymal stem cell populations that contribute to maintaining stemness and are associated with chemoresistance [52,53]. Raghavan et al. demonstrated that the carcinoma-associated mesenchymal stem/stromal cells enhanced stem-like features via secretion of platelet-derived growth factor (PDGF), and that knockdown of PDGFB resulted in improved sensitivity to platinum [53]. Exposure to standard-of-care chemotherapies or PARP inhibitors enriches CSC populations [54,55], highlighting the challenge that CSCs pose to effective treatment and development of therapy resistance.

A recent study by Robinson et al. aimed to characterize the roles of SOX2, OCT4, and NANOG in ovarian CSCs [56]. Following sequential carboplatin treatment of 2D cultured ovarian cancer cell lines, only SOX2 expression remained elevated after the third treatment, suggesting a role for SOX2 in platinum resistance [56]. Knockdown of SOX2 reduced spheroid formation and improved platinum sensitivity [56]. Additionally, while the presence of traditional CSC markers (CD117, CD133, ALDH1) was variable across cell lines, SOX2 was consistently increased in 3D conditions, and elevated in recurrent cases of ovarian cancer, suggesting it may be a more reliable functional marker of CSC and disease recurrence. Imaging and sequencing analysis of fallopian tube epithelium from HGSOC patients and control group from patients with benign conditions as well as BRCA1/2 germline mutation carriers strongly suggested that SOX2 is transitionally upregulated in the early stages of the disease development and could also be an interesting candidate as a potential biomarker of early disease [57].

### 2.4. Clonal Evolution and Changes in Cellular Mechanisms at Different Stages of Disease Progression

A widely accepted postulate of molecular carcinogenesis is that the vast majority of solid cancers have a monoclonal origin, where a single event of cellular transformation is followed by a sequence of events and progression based on the competitive advantage of the cancer tissue at the expense of healthy tissue. Molecular divergence of malignant tumours during disease progression is extensive, and tumour heterogeneity is particularly prominent in aggressive and fast-growing types such as HGSOC. An increase in heterogeneity is thought to be driven by differences in selection pressures within local microenvironments at distinct sites. Moreover, treatments by chemotherapy agents foster the emergence of resistant clones, and disease recurrence is still an inevitable long-term outcome for most patients. Different growth requirements for evolving cancers can lead to a reversal of phenotypic characteristics at times, as metastatic cancer cannot be considered as a solely unidirectional process of loss of differentiation and continuous reduction in fidelity of cell functions. There are several examples of genomic alterations, which are essential for cell transformation, that are reverted at later stages of the disease. This phenomenon is well documented in the cases of recurrent chemoresistant ovarian cancer which has been shown to reacquire DNA repair proficiency by reversion of BRCA1/BRCA2 mutations or modification of epigenetic marks [58,59]. Moreover, the role of Wnt signalling in communication between stroma and the tumour likely alters during the disease progression.

A large genome-wide association study (GWAS) and meta-analysis of sporadic epithelial ovarian cancer cases (15,437), BRCA1 and BRCA2 carriers (15,252 and 8211 respectively) and 30,845 healthy controls identified genomic variants of Wnt pathway components, Wnt4 and RSPO1 genes, as new susceptibility loci [60]. Analysis of this large data set suggests that even subtle defects in Wnt signalling activity in the fallopian tube epithelium increase the propensity of the epithelium to transformation events. In contrast, there is evidence that active Wnt signalling plays an important role in the development of platinum resistance in advanced HGSOC [61,62].

Regulation of the processes of differentiation at the SCJ in the cervix is central to the understanding of the carcinogenesis of this cancer type, which is an HPV-driven event, but occurs very rarely as the vast majority of viral infections are resolved without long-lasting impact. Which combination of intrinsic and broader microenvironmental factors triggers expansion of the latent HPV-infected cells requires further investigation, but in this case, it is also clear that selection and clonal evolution are likely dependent on the broader tissue context.

## 3. Organoids Recapitulate the Main Characteristics and Tissue Hierarchy of Epithelial Tumours In Vitro, and Are a Potential Tool for Personalisation of Patient Therapy

Organoids are an attractive prospect for personalising patient treatments because it is now well established that patient-derived organoids (PDOs) resemble the parental tumour, as confirmed by genome sequencing and phenotypic analysis, and are applicable to drug screening [63]. Tumour ex vivo explant culture has also been established for different cancers and commercialized, with tailored TME ecosystems, to conserve patient/tumour heterogeneity and offer personalized therapeutic options for patients [64,65,66,67]. However, despite offering physiological, ethical, and financial advantages, explant cultures have a very short window of opportunity for therapeutic screening. In contrast, PDOs have multiple advantages such as self-renewal and expansion to test multiple drugs/combinations, capable of long-term storage for bio-banking and future regeneration for drug screening, and suitability for molecular genetic manipulation. Analogous to organoid formation capacity and in vitro differentiation potential of stem cells from the healthy epithelium from the fallopian tube, endometrial glands, and cervix epithelium, malignant tumours of the genital tract also contain stemness potential that drives developments of cancer organoids in vitro. Longevity of stem cells which drive expansion of organoids in culture and continuous differentiation make organoids adequate models to test in vitro both strategies that have been previously described as viable approaches to eradicate CSCs [68], by direct targeting of sternness potential and forced differentiation.

A recent study establishing HGSOC PDOs from chemo-naïve tumours identified a shift in required stem cell growth conditions for HGSOC, in contrast to healthy Fallopian tube organoids, and detected that key regulatory changes in markers of stemness and differentiation occur early in the development of HGSOC tumourigenesis [69]. However, in advanced HGSOC, evolution in the cellular mechanisms which preserve CSCs potency remains elusive, and our knowledge about molecular origins of chemoresistant clones is rudimentary. Little is known about the clonal evolution of CSCs from primary disseminated disease to recurrent HGSOC, the presence of CSC populations within relapse tumours, and how they relate to the initial stem cell population in the primary tumour.

Propagation of PDOs to determine drug or radiotherapy efficacy is becoming more commonplace for different gynaecological malignancies. In particular, significant progress has been made for establishing drug treatment parameters and platforms for PDOs derived from EOC tumours, albeit currently at a low-medium throughput level. PDOs from EOC cases were treated with standard-of-care first-line chemotherapies (carboplatin, cisplatin, paclitaxel) and relevant targeted therapeutic agents (PARP inhibitors, PI3K inhibitors), and responses recorded correlated with the patient’s clinical responses [63,70,71,72]. A direct comparison of drug screening in 2D ovarian tumour cells and ovarian cancer PDOs demonstrated that cytostatic drug efficacy differs between the two culture systems, linking organoid drug sensitivity to DNA repair deficiency in the PDOs, findings not noted in the 2D monolayer cultures [72]. Moreover, PDOs revealed inter- and intra-patient heterogeneity in responses to drug treatments for a small number of patients [63,70], strongly illustrating that subsequent studies must include further sites of tumour dissemination to fully characterise the intrinsic heterogeneity existing within patients with EOC and accurately model tumour therapeutic responses. A potential limiting factor of a number of previous studies on EOC organoids is the propagation of organoids from neo-adjuvant tumours [63,70]; these tumour samples may already exhibit biological variations due to pre-treatment with chemotherapy [69].

Studies which established human endometrial PDOs include the optimization and differentiation of hormone-responsive organoid cultures. Furthermore, upon exposure to pregnancy signals, endometrial organoids displayed characteristics of early pregnancy [73]. Organoids mimicked the normal physiological responses of the endometrium to hormonal control, e.g., oestrogen promoted increased cell proliferation, and additionally, hormone treatment of human endometrial organoids allowed replication of the menstrual cycle [28]. Single-cell RNA-sequencing was employed to create a high-resolution gene expression atlas of endometrium organoids, and provided information on their responsiveness to hormone treatment (oestrogen and progesterone), replicating gene expression changes in proliferative and secretory phase endometrium [74]. PDOs have also been established from endometriosis, precancerous states such as endometrial hyperplasia and Lynch syndrome as well as low and high-grade endometrial cancers [29]. PDOs from these endometrial diseases demonstrated long-term expansion properties, transcriptomic and genomic stability, and captured the clinical heterogeneity of each condition and disease setting. Endometrial cancer organoids were amenable to drug screening of standard chemotherapies, demonstrating patient-specific drug responses, in particular sensitivity to mTOR inhibition in line with mutations in the PI3K/AKT/mTOR signalling pathway [29].

Recent comprehensive studies deriving PDOs from cervical cancer cells or normal cervical tissue have been described. Organoids have been developed from normal cervical cells from the SCJ region, and metastatic squamous cells from the transformation zone, providing models in which HPV-driven cervical carcinogenesis could be evaluated [75]. Furthermore, organoids established from human and mouse ecto- and endo-cervical cells revealed that the two epithelial cell types descend from distinct cervical lineage-specific stem cell populations which are regulated by divergent stromal Wnt signals. PDOs from human ecto- and endo-cervical lineages faithfully recapitulated the in vivo architecture and could be maintained in culture for more than six months [12]. Radiation therapy is a mainstay therapy for cervical cancer patients, and the radiosensitivity of cervical cancers is quite diverse. Radiotherapy treatment of organoids could be an alternative approach to predict radiation sensitivity of patient tumours. Different groups have attempted to interrogate the radiosensitivity of cervical tumour organoids and spheroids to investigate mechanisms underlying radiation resistance. Inhibition of growth of small cell carcinoma of the uterine cervix (SCCC) organoids was observed in a dose-dependent manner post-irradiation, and variable radiosensitivity profiles were detected across PDOs from individual patients. Radiation-induced upregulation of HIF-1α in radioresistant SCC organoids was proposed as a mechanism of radioresistance [76]. Organoids derived from another rare subtype of cervical cancer, clear cell cervical cancer (cCCC), were found to resemble the tumour of origin and demonstrated chemosensitivity following treatment with common chemotherapy agents and MET inhibitors [77].

In addition to a necessary focus on cancer driving genes and developmental pathways which control epithelial homeostasis, it is becoming clear that micro-RNAs and noncoding RNAs could regulate CSC potential on a post-transcriptional level. Micro-RNA family miR-34 has been described in several malignancies as tumour-suppressive and thus could be a promising pharmacological therapeutic candidate [78]. As TP53 is the main regulator of miR-34 expression, this could be of great interest for HGSOC treatment [79]. Indeed, a recent study found significantly lower levels of miR-34 in HGSOC tumours in comparison to low-grade ovarian cancer tissue, in line with differences in TP53 mutation status [80].

### 3.1. Homologous DNA Repair in the Context of Stem Cell Biology of the Genital Tract

Potential mechanistic links between cell cycle regulation and DNA homologous recombination machinery and mechanisms which control stemness and differentiation warrant further investigation. Moreover, better insight into the involvement of chromatin remodelling complexes in the development of gynaecological malignancies is required. NGS studies revealed frequent mutations in ARID1A in the endometrioid type of ovarian cancer as well as in endometrioid adenocarcinoma of the uterus [81]. ARID1A is a member of the chromatin modelling complex SWI/SNF complex. Interestingly these mutations are mutually exclusive with TP53 in endometrial cancer; thus, ARIDA1 identified only cancers which are TP53 wild-type.

HGSOC, on the contrary, is driven by mutated TP53 gene, and in almost 50% of cases, homologous DNA repair is deficient, due to germline mutations, somatic mutations, or epigenetic inactivation of BRCA1/ BRCA2 genes. Tumours with impaired HR have a high sensitivity to platinum-based chemotherapies. Recent clinical studies have established the benefit of PARP inhibitor therapies in patients who have homologous recombination deficiency (HRD) even in the absence of BRCA1/2 mutations [82,83,84], though the predictive value of HRD scores remains limited. While emerging resistance to PARP inhibitors complicates their expanded usage and requires further improvements in patient stratification, BRCAness is a positive prognostic factor for HGSOC patients [85]. High prevalence of cyclin E amplification and RB1 pathway signalling activation are identified as molecular markers of tumours resistant to platinum and in recurrent disease [86]. Interestingly BRCA1/2 mutations and CCNE1 amplification are mutually exclusive events [87] which is an indication that DNA repair proficiency is essential in cancer cells where phase G1 to S phase transition is deregulated and there is increased chromosomal instability.

### 3.2. Application of HRD Screening in PDOs

Due to nearly half of HGSOC cases demonstrating deficiency in homologous recombination repair, screening for HRD status to inform on treatment options has entered clinical routine. For example, tests have been developed based on genomic footprints to identify HRD to predict the use of PARP inhibitors (PARPi) such as the myChoice CDx [88], Classifier of Homologous Recombination Deficiency (CHORD) [89], or Foundation Focus CDx _BRCA_ test [90]. Nevertheless, clinical practice has repeatedly shown that prediction of patient response to PARPi solely based on the analysis of the mutational profile is not optimal, and should be complemented with robust in vitro readouts of HRD proficiency [91]. A functional HR assay, the recombination Repair CAPacity (RECAP) test, was originally developed to identify HRD in breast cancers [67,92] and more recently ovarian cancers [93], detecting both BRCA1/2-deficient and BRCA1/2-proficient HRD tumours in ex vivo tumour slice cultures. This type of functional assay, detecting the formation of RAD51 foci in proliferating cells following DNA damage, is applicable to organoid screening and was recently tested in HGSOC organoids from a small number of patients to predict PARPi sensitivity [63]. Another study comparing whole-exome sequencing (WES) data and RAD51 foci formation capacity and in vitro response to olaparib in 33 short–term organoid lines from 22 patients demonstrated that incidence of PARPi resistance is much higher than HRD signatures predict (2 out of 12 lines were sensitive to olaparib) [71]. These important findings underline the need to extend and further optimise functional in vitro assays for HRD detection to improve stratification of patients regarding PARPi treatments.

### 3.3. Translational Application of Organoid Models in Clinical Trials

At present, there are fifty-three clinical trials registered (active, recruiting, or not-yet recruiting) with clinicaltrials.gov (accessed on 10 May 2021) to evaluate PDOs as representative models of cancer for screening of different therapies, seven of which relate to gynaecological malignancies, mainly ovarian cancers. Prospective trials of colorectal cancer PDOs demonstrated a correlation of patient responses to chemotherapy and targeted agents with ex vivo PDO drug screening [94,95]. Nevertheless, to establish PDOs as a viable clinical tool to assess drug responses to offer treatment options, PDO propagation and screening should be performed within a short time interval from the time of diagnosis or relapse to be clinically beneficial and coupled with rapid genomics testing. Recent studies have demonstrated a feasible turnaround time of 3 weeks from tumour cell extraction to data collection from formed PDOs [63,70,71,72,96]. This proposed timeline was improved using a ring-based format for quicker PDO formation and drug screening of EOC organoids in a high-throughput system, with results available within a week from the time of surgery [97]. The broader implication of establishing clinically-relevant PDO models for routine testing of therapeutics and capturing the complex heterogeneity of individual patients, is that it should lead to development of better therapeutic strategies and personalisation of therapy to benefit women with complex malignancies, thus preventing them from being exposed to unnecessary iatrogenic toxicity.

## 4. Future Perspectives

### Applications and Novel Engineering Approaches for PDOs

**Figure 2:**
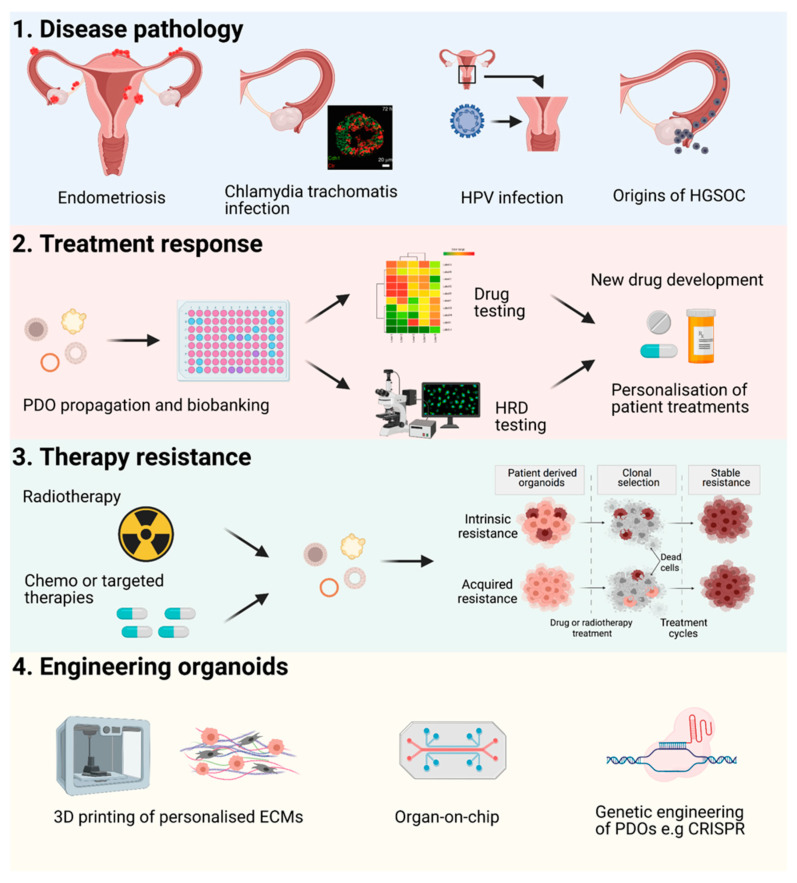
Overview of the applications of organoids in the research of gynaecological malignancies. 1. Organoids can model different cell processes and disease pathology such as endometriosis, bacterial infections such as *Chlamydia trachomatis*, HPV infection leading to cervical cancer, and investigating the origins of HGSOC. 2. PDOs can also be propagated for long-term biobanking for future regeneration. PDOs are amenable to drug screening to mimic patient drug responses to guide personalisation of therapy or improve pharmaceutical drug discovery rates. Assays to assess homologous recombination capacities of PDOs can also be performed. 3. PDOs can also be used to model cancer progression and development of resistance to standard-of-care patient therapies. 4. Future applications for engineering of organoids to improve derivation rates include 3D printing of tailored extra-cellular matrices, introducing fluid flow with microfluidics or organ-on-chip technologies. PDOs can also be genetically engineered for example via CRISPR technology to mimic disease genomes.

The applications of human organoids are numerous from precision medicine to disease modelling (Figure 2). They provide basic models for research to uncover and model developmental processes and basic cell-to-cell interactions. Significant advantages of organoid models over ex vivo explant models include the potential for long-term propagation or differentiation of organoid cultures and biobanking of PDOs for future regeneration for patient drug screening, e.g., in a clinical trial setting or future research. PDOs are also potential tools for regenerative medicine coupled with genetic engineering [98]. Within these many future applications of organoids, there is also scope for improvement of PDO derivation and culture rates by applying engineering approaches to various levels in organoid growth, development, and drug assay read-out or imaging (for a detailed review see Hofer et al. [99]). Cell–cell and cell-matrix adhesion, genetically engineered fate choices could be applied to modify cells for organoids. Tissue/tumour microenvironmental factors could be modified or personalised for organoid context through the use of more applicable hydrogels tailored with different “designer” ECM compositions or decellularized ECMs to factor intra-tumoural matrix heterogeneity into organoid cultures, control of ECM stiffness, and modifications of cell media with different growth factors and metabolites [100]. Control of fluid flow via microfluidics or organ-on-chip methodology could provide further options for the development of organoid systems [101]. Different organ-on-chip systems have already been trialled for stomach [102] and kidney [103] organoids, introducing fluid flow into these models. Maximising read-out options for functional assays for drug screening could also benefit from new technological approaches. Improved image-based analysis to enable setup of robust high-throughput drug screening, fully integrated with microfluidics platforms for uniformity of firstly growth of large numbers of PDOs in a 96 or 384-well format and screening of multiple drugs and drug combinations is necessary to maintain consistency of drug treatments in functional assays, data capture and subsequent analysis for personalisation of patient treatments or drug discovery applications.

To further recapitulate the in vivo physiology of the tumour and surrounding stroma and cell constituents, efforts have turned to incorporation of other important cell types to create co-culture organoids, with significant strides made in other cancer types developing co-cultures with immune cells [104] and/or cancer associated fibroblasts [105,106,107,108]. Limited work to date has been done on co-cultures of genital tract tissue or cancer PDOs as part of broader efforts to establish 3D cultures to include the wider immune microenvironment. However, a recent study investigating simultaneous PD-1 and PD-L1 immune checkpoint blockade in HGSOC developed novel HGSOC organoid/immune cell co-cultures to determine the efficacy of immune therapies [109]. To fully recreate the tumour microenvironment to accurately model PDO drug responses for personalisation of patient treatments, efforts must continue to incorporate stromal and immune cells in co-culture systems to promote cross-talk enabling crucial intracellular signalling between cells. This approach would help to build upon unique advantages of PDOs in comparison to other valuable disease models such as patient derived xenografts (PDXs). The latter are still considered as “gold standard” to assess tumourigenic potential of any cancer type, and have been successfully created for gynaecological malignancies [110,111]. However, despite providing clear benefits as in vivo experimental models [112], PDXs do have limitation of xeno-tissue environment. This could be especially significant in efforts to characterize interactions between cancer and innate and adaptive immune system, both of which have significant differences in mouse and human.

## 5. Conclusions

### Clinical Perspective

Taken together, recent advances in our understanding of changes in stem cell regulation and epithelial homeostasis during carcinogenesis and disease progression have opened promising new approaches in the research of gynaecological malignancies, with great translational potential. Cancer stem cells appear be able to modulate core signalling pathways in epithelial ovarian cancer and are believed to be responsible for disease progression, relapse, and drug resistance development [113]. Hence, cancer stem cells appear to play a key role not only in carcinogenesis but also in the natural history of the disease and contribute to its heterogeneous profile. They represent a promising therapeutic platform that, once decoded, can potentially open numerous possibilities for the resolution of this challenging cancer entity [1]. Evidence has demonstrated cancer stem cells induce and influence progression or relapse and are characterized by a rather slow-cycling rate which makes them resistant to standard cytotoxic treatments [113,114,115,116]. Presumed hypotheses suggest that the high probability of relapse of advanced epithelial ovarian cancer is possibly attributed to a subpopulation of quiescent epithelial ovarian cancer stem cells that, by remaining in the G0 phase of the cell cycle, are not sensitive to cytotoxic treatments. However, once they return to an active reproduction phase, they can become the potential driving force of the cancer relapse [113]. Direct correlations between the onset of chemoresistance and the abundance of CSCs is an emerging field and requires further exploration and validation [116,117,118]. In addition, long-term follow-up and functional analysis of PDOs derived from patients at different stages of disease progression could help identify mechanisms of cellular perturbations that drive tumour growth in response to therapy.

A further perspective for exploration is the field of early diagnosis and detection of CSCs. Many recent technologies have focused on the endoscopic or hysteroscopic examination of the fallopian tubes and/or uterine lavage fluid with clinical trials ongoing to test these devices [119]. These technologies are based on the principle that cells from HGSOC, or precursor STIC lesions, can exfoliate and be secreted together with the endometrial fluid, which in turn can be easily accessed, representing a promising avenue for earlier diagnosis [119]. Although currently, collection of these cells is challenging and not yet routine, it is a highly promising emerging approach that could be implemented into clinical practice once the technology is fully optimised. We could envisage the development of organoids from the cells isolated from uterine lavage that can in turn be used for profiling the biology of the disease and to predict patients’ response to treatment.

Overall, it can be concluded that complex 3D patient-derived organoid culture models provide new and versatile experimental platforms to study gynaecological malignancies. They create opportunities to identify central mechanisms of cancer biology, which remain poorly understood such as tissue hierarchy and clonal diversification, influence of microenvironment and interactions with immune system. This could lead to development of new, better-tailored therapeutic concepts that help towards achieving the long-term goal of improving detection and patient outcomes.
